# Technostress and exhaustion as threats to work engagement, examining the protective role of technology self-efficacy

**DOI:** 10.3389/fpubh.2026.1897279

**Published:** 2026-09-07

**Authors:** Dagmawit Dansa, Cheasley Lentor, Martha Harunavamwe, Herbert Kanengoni

**Affiliations:** 1 Department of Human Resource Management, Faculty of Economic Management Sciences, University of Pretoria, Pretoria, South Africa; 2 Department of Industrial Psychology and People Management, College of Business and Economics, University of Johannesburg, Johannesburg, South Africa

**Keywords:** banking industry, exhaustion, technology self-efficacy, technology work demands, technostress, work engagement

## Abstract

**Orientation:**

The digitization of the banking industry has intensified employees' exposure to technology related work demands, heightening the risk of technostress and exhaustion. The study investigates the relationship between technostress and exhaustion examining the moderating and mediating role of technology self-efficacy in sustaining work engagement within the banking industry.

**Research purpose:**

This study seeks to understand whether technology self-efficacy can be seen as a protective personal resource that may buffer the effects of technostress and exhaustion and sustain work engagement.

**Motivation for the study:**

Integration of technology has increased employees' exposure to technostress and exhaustion, posing threats to work engagement. Therefore, the study examines the protective role of personal resources in mitigating the negative effects of technostress and exhaustion on work engagement to maintain productivity in digital environments.

**Research approach/design and method:**

Adopting a positivistic paradigm, this quantitative, cross-sectional research collected data from 202 banking employees via Qualtrics. Data were analyzed using the variance based Structural Equation Modeling (SEM) specifically SmartPLS.

**Main findings:**

The findings confirms that technostress simultaneously increases exhaustion and reduces work engagement indicating the dual role as both a strain inducing demand and a motivational inhibitor. Technology self-efficacy and exhaustion partially mediate the relationship between technostress and work engagement. Though technology self-efficacy supports work engagement, it does not prevent exhaustion, thus it cannot fully offset fatigue under high technology demands.

**Practical/managerial implications and contribution/value-add:**

Banks should prioritize digital skills development alongside workload and wellbeing interventions. Sustaining work engagement in modern environments requires a combined approach of reducing techno stressors, promoting employees' digital capabilities while strengthening employee recovery to prevent exhaustion and disengagement.

## Introduction

In modern organizations, technology has become an important tool for enhancing efficiency, communication and productivity (1, 2). However, the rapid integration of technologies has also introduced new challenges for employees. One of the most significant challenges is technostress, a form of stress that arises when individuals struggle to cope with technological demands, constant connectivity, information overload and continuous need to adapt to new systems and applications (3). Recent systematic reviews demonstrate that technostress has become a pervasive occupational health concern associated with adverse employee wellbeing and negative work outcomes, highlighting the need for both individual and organizational coping mechanisms (3, 4). Research also indicates that technostress negatively influences employees' work engagement in hybrid and virtual work settings, particularly when compounded by work-family conflict and inadequate organizational support (5). Despite the adverse consequences of technostress and exhaustion, employees do not all respond to these demands in the same way. One important personal resource that may help individuals to cope with technological demands is technology self-efficacy defined as an individual's belief in their capability to successfully use and manage technology (6). Employees with higher levels of technology self-efficacy are generally more confident in learning and using new technologies, demonstrate greater adaptability to technological change and are better equipped to overcome technology related challenges.

Drawing on the Job Demands Resource Model (JDR), technology self-efficacy can be seen as a personal resource that mitigates the negative effects of job demands (7). Technology self-efficacy may serve as a protective mechanism that buffers the adverse effects of technostress and exhaustion on work engagement. Employees who possess strong confidence in their technological capabilities are more likely to maintain positive attitudes, experience lower levels of strain despite increasing technological demands and remain engaged in their work, even when confronted with increasing technological challenges (5). Understanding this protective role is particularly important as organizations continue to undergo digital transformation and seek ways to sustain employee wellbeing and performance.

With the above background in mind, it is clear that rapid digitalisation has transformed work processes, imposing heightened physical and psychological demands on workers (8). The increasing reliance on information and communication technologies (ICTs), such as smartphones, laptops, email, and instant messaging, has introduced various stressors (9). Kumar et al. (10) noted that modern organizations stand on the brink of even greater technological innovations, poised to revolutionize how work is performed and how technology is applied in a way that sustains engagement and productivity. As the digital workplace continues to evolve, understanding the consequences of technostress becomes crucial for organizations aiming to foster a healthy and sustainable work environment (11). This involves not only identifying the negative impacts of technostress, such as exhaustion, but also recognizing its potential impact on positive outcomes such as work engagement (12).

Though technology affords employees considerable power and possibilities, the reality for many users is one of difficulty and feelings of being overwhelmed, with devices and applications leading to anxiety, overload, addiction, distraction, exhaustion, and burnout (13). Research has indicated that increased work demands due to technological advancements can lead to feelings of overwhelm and burnout among employees (11). Similarly, inadequate technological proficiency may result in frustration and decreased job satisfaction (14), as well as role ambiguity and uncertainty about job responsibilities leading to stress and decreased productivity (100). Additionally, the intrusion of technology into personal lives can blur the boundaries between work and home life (15), and due to concerns about job security, individuals persistently experience feelings of anxiety (11).

While technostress has impacted various industries, evidence indicates that the banking industry has been disproportionately affected. The contemporary banking industry operates in a highly complex and competitive environment, shaped by rapid technological innovation, digital disruption and shifting consumer demands (16–18). Jenkin and Naude (19) noted that digital banking, which enables financial services through online platforms, has increasingly supplanted traditional branch-based banking, borderless marketplaces and is forcing institutions to adapt their operations, products and service channels to remain relevant, which is further supported by Mothibi and Rhulani (20). In South Africa, leading banks have implemented swift innovation and digitalisation strategies, including hybrid models to meet these evolving demands (21). While technology provides major operational benefits, constant digital engagement and updates require continuous connectivity, which has notable drawbacks (22, 23).

In the banking set-up, work is technology-intensive and there is prolonged exposure to digital tools, resulting in fatigue, cognitive overload and emotional exhaustion, undermining work engagement (24). Similarly, research has demonstrated that excessive use of technology is positively associated with employee exhaustion and work engagement, as individuals experience difficulties adapting to new devices and software applications (5, 25). In particular, the combination of excessive use, limited familiarity with digital tools and uncertainty regarding emerging technologies contributes to technostress, which constitutes a critical mechanism through which exhaustion erodes work engagement. Employees who are unfamiliar with specific tools or systems may experience heightened stress that accelerates burnout, which in turn further undermines their capacity to remain engaged at work.

Given the challenges associated with technostress and its detrimental effects on work engagement as well as its potential to culminate in exhaustion, managers are increasingly seeking effective strategies to mitigate technology-induced strain (13). While coping strategies help, Tarafdar et al. (26) emphasize that more comprehensive and people-centered approaches are necessary to bolster technological confidence and resilience in the digital workplace. With technology now central to organizational operations (27), recognizing the role played by personal resources has thus become crucial. Research has indicated that employees with higher technology self-efficacy are more likely to approach technological challenges with resilience, adapt to new systems efficiently, and recover better from stress-induced exhaustion (5, 27, 28). However, personal resources, particularly strong technological self-efficacy, can assist with these challenges (27). This study explores the relationship between technostress, exhaustion, and work engagement, specifically looking at the role played by technology self-efficacy within the banking industry.

## Literature review and hypothesis development

### Technostress

Technostress refers to the stress an individual experiences due to their use or overuse of technology at work (1). Technostress refers to difficulties encountered in adapting to the requirements of information and communication technology (ICT). It consists of five sub-dimensions: techno-overload, techno-invasion, techno-complexity, techno-insecurity, and techno-uncertainty (1). Techno-overload describes situations where technology leads employees to work at an increased pace, while techno-invasion refers to expectations of constant availability (29). Techno-complexity is the stress caused by finding recent technologies hard to grasp, while techno-uncertainty stems from constant system changes that hinder employee mastery. This uncertainty may also lead to techno-insecurity, reflecting fears about one's competence or job stability in the face of evolving technological requirements. With hybrid models becoming the norm, the intensity of technology use has only grown, making technostress one of the more urgent stressors modern employees may encounter (29).

### Technostress and work engagement

With modern workplaces increasingly relying on digitized tools and platforms, the potential for technostress to impair work engagement has increased. Kahn (30) defines work engagement as the act of fully investing oneself in work, while Schaufeli et al. (31) offered a more widely adopted view, defining it as a positive, fulfilling work-related state with three elements: vigor, dedication, and absorption. In the modern workplace, work engagement has gained increasing traction due to its link with better performance, commitment, and job satisfaction (32–34). Engaged employees tend to be more productive, emotionally invested, and less likely to quit, while exhausted employees often show low morale and reduced output (35). Employees today must adapt to new digital systems and manage their core duties, often with little training or support. This may result in detachment and emotional exhaustion (32).

Recent studies suggest that the demands of navigating multiple digital platforms simultaneously, including apps, collaborative software, and automated reporting tools, create persistent strain that diminishes employees' capacity to remain fully engaged (5, 25). Technostress is associated with negative emotions such as skepticism, inefficiency, mental tiredness, and disengagement (24). In universities, technostress increases exhaustion and workload, leading to fatigue and lower motivation, which reduces employee engagement (36). This suggests that technostress may negatively affect an individual's ability to perform optimally at work. However, findings are mixed. While some studies suggest technostress reduces engagement, others reveal the opposite. Tarabay (35) found a positive correlation between technostress and work engagement in the Lebanese labor force, while Okolo et al. (18) observed that engaged employees in the banking sector may better cope with technostress. These contrasting results highlight the need to further explore the nature of this relationship, particularly in banking contexts.

*H1: technostress has a negative and statistically significant direct effect on work engagement*.

### Technostress and exhaustion

In the workplace context, exhaustion refers to feeling emotionally and physically overextended due to prolonged demands (37, 38). This study defines work exhaustion as the depletion of emotional and mental resources required to meet workplace responsibilities (39). It includes emotional, physical, and cognitive exhaustion, which together reflect the layered nature of burnout (37, 40). In adapting to continual technological changes and maintaining the skills required to operate in high-technology platforms, employees are bound to experience heightened strain that can progressively lead to exhaustion or burnout. Exhaustion emerges from ongoing exposure to high job demands, often leading to a state of emotional and physical depletion (41). This is especially common among employees who interact with technology and roles that require extensive use of technology; thus, these roles experience mental fatigue from constant digital interaction (41). Exhaustion manifests as reduced motivation, fatigue, and diminished accomplishment, with contributing factors including overload, underload, and prolonged stress (42). 40 emphasize that continuous exposure to new technologies can negatively affect mental health.

*H2: technostress has a positive and statistically significant direct effect on exhaustion*.

### Technology self-efficacy as a mediator in the relationship between technostress and work engagement

Given the escalating pressures of technostress in modern digitized workplaces, personal resources such as digital literacy and technology self-efficacy play a crucial role in preserving work engagement. Individuals with high technology self-efficacy tend to have more positive work experiences and better psychological outcomes (28). Bandura's self-efficacy theory suggests that belief in one's ability to succeed shapes motivation, effort, and emotional response (32, 43, 44). Self-efficacy develops from mastery, observation, encouragement, and emotions. In the context of technology, this refers to one's confidence in using digital tools effectively for work tasks. While early studies called it “computer self-efficacy” (6), more recent research defines technology self-efficacy as the belief in one's competence to use ICTs, shaped by experience, confidence, and task-specific skills (39).

Technology, while streamlining tasks, can increase job complexity and affect work-life balance (45). However, when technology self-efficacy is high, these negative effects can be reduced. Thus, evidence indicates that employees who feel confident in using ICTs are more likely to perceive technological demands as manageable rather than overwhelming. Technology and computer self-efficacy are associated with greater engagement, and higher digital literacy can reduce negative effects. es self-efficacy, which enables employees to better manage their technology job demands and reduce burnout risk while maintaining work engagement (46). Fostering a digital mindset, including a positive and flexible orientation toward digital tools, can moderate negative responses to technostress, improving work engagement and job satisfaction even when the stressors are present (47).

Recent findings in a university setting revealed that positive ICT adoption completely mediates the relationship between technostress and work engagement, alluding that increased levels of technology self-efficacy may lead to easy ICT adoption, which in turn improves work engagement (48). Thus, technology self-efficacy as a personal resource, from the JD-*R* model perspective, can influence positive work outcomes, including work engagement and wellbeing (28). Technology self-efficacy is an individual trait that supports meeting technology-related job demands. By enhancing employees' ability to cope with and adapt to digital change, technology self-efficacy mitigates the adverse effects of technostress, thereby reducing the risk of exhaustion while sustaining engagement. Therefore,

*H3: technology self-efficacy mediates the relationship between technostress and work engagement*.

### Technology self-efficacy as a mediator in the relationship between technostress and exhaustion

Shen and Kuang (49) found that the demands of workplace ICT can drain psychological resources, leading to exhaustion and even behaviors like knowledge hiding. When technology pressure builds up, energy drops, and wellbeing suffers (38), thus technostress depletes energy and results in exhaustion, which is often the first sign of that strain. Research indicates that continual technological change can increase job demands and is associated with outcomes such as burnout and detachment (41). Within this context, technology self-efficacy functions as a critical personal resource that enables employees to manage technology-related stressors with greater confidence and adaptability. Ma et al. (45) revealed that technology self-efficacy significantly weakened the relationship between technostress and emotional exhaustion among teachers. A more recent study demonstrated that mobile self-efficacy buffered the negative impact of stress on academic engagement (38). Consistent with that, Gemmano et al. (50) noted that techno-overload, invasion, and complexity disrupt the work-family balance and lead to greater exhaustion. According to the JD-*R* model, exhaustion arises when job demands like technostress outweigh available resources. And as Bandura's theory reminds us, believing in one's technological abilities (self-efficacy) is key to coping and staying motivated (51). From the Conservation of Resource (COR) theory (52), self-efficacy serves as a valued resource that protects individuals against resource depletion and prevents the downward loss spirals commonly associated with technostress. Therefore,

*H4: technology self-efficacy mediates the relationship between technostress and exhaustion*.

### Technology self-efficacy as a moderator in the relationship between technostress, exhaustion, and work engagement

Technology self-efficacy may not only function as a direct personal resource but also as a critical moderator in the relationship between technostress, work engagement, and exhaustion. According to COR theory (52), individuals with higher technology self-efficacy are better able to protect their resources and avoid loss when facing technology-related challenges. Zhang and Chen (53) showed that digital self-efficacy reduced the detrimental effects of technology burnout on engagement, highlighting its potential to sustain motivation and energy. This suggests that technology self-efficacy may function as a protective mechanism, mitigating the draining effects of technostress on employee wellbeing and sustaining engagement. Similarly, evidence shows that digital self-efficacy reduces burnout and enhances satisfaction with technology use, eventually positively contributing to work engagement (38). In addition to that, Ali et al. (24) highlighted how computer self-efficacy moderates the relationship between technostress and workload, which in turn affects work engagement, and that employees with stronger self-efficacy experience less anxiety and better performance. Kim and Lee (54) extended these findings to the IT sector, showing that self-efficacy and support reduce counterproductive behaviors and resistance to innovation.

In addition, based on the JD-*R* Model, personal resources such as self-efficacy enable employees to manage stressors more effectively, thereby sustaining motivation and engagement despite the demands (55). In technology-driven environments, employees with higher levels of technology self-efficacy are more confident in navigating digital demands, which may reduce the depleting effects of exhaustion and foster sustained engagement (56). Research indicates that self-efficacy may influence how job stressors relate to engagement. Employees with higher confidence in their technological skills can experience more favorable work outcomes when facing pressure (57). Therefore, it is plausible to hypothesize that

H5a: *technology self-efficacy moderates the relationship between exhaustion and work engagement*.

Again, from the JD-*R* model perspective, technostress is a job demand, while technology self-efficacy is a personal resource that helps buffer these demands and maintain engagement. Gaudioso et al. (58) revealed that high levels of technology self-efficacy assisted employees in better coping with daily demands, reducing exhaustion and maintaining engagement. Employees with higher technology self-efficacy perceive technology-related challenges as manageable rather than overwhelming, which reduces strain and promotes positive motivational outcomes (56). It enables employees to adapt to technological demands with greater resilience, protecting them from disengagement. Prior research highlights technology self-efficacy as a key resource that mitigates the negative impact of technostress and helps to maintain engagement in technology-intensive environments. Rayburn et al. (59) found that technology training can improve technology self-efficacy, improve technology adaptation and performance, while Yener et al. (27) also indicated that self-efficacy reduces techno-overload, techno complexity, and techno-insecurity as well as communication fatigue. Therefore, we hypothesize that

*H5b: technology self-efficacy moderates the relationship between technostress and work engagement*.

**Figure 1:**
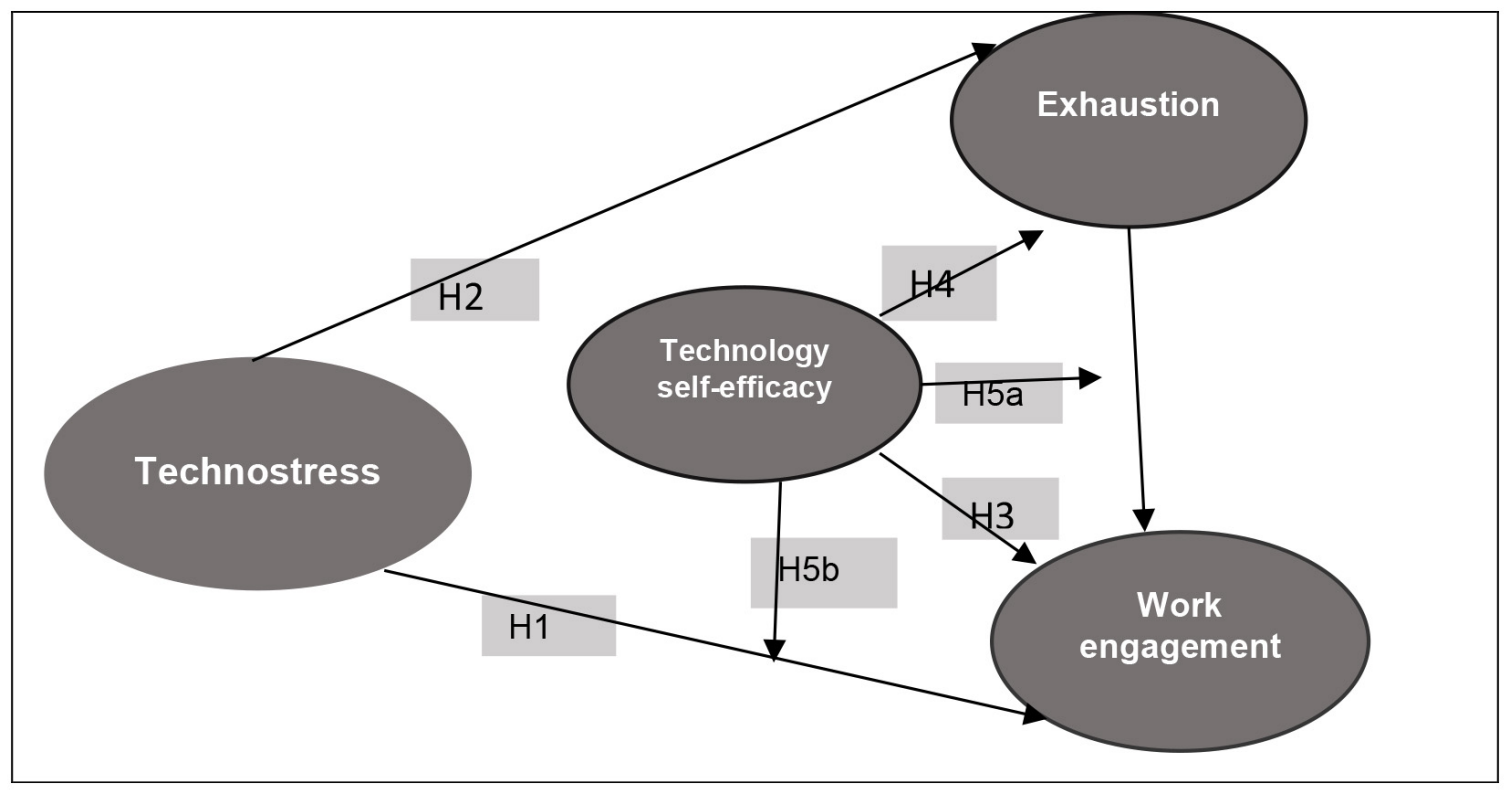
Conceptual Model.

Figure 1 below illustrate the conceptual framework and the hypothesis based on the literature presented above.

### Theoretical framework

The JD-*R* model explains how job demands like technostress drain employees over time, especially when those demands involve constant digital pressure or mastering complex tools (7, 60). When job demands exceed employees' capacity to cope, exhaustion can result, leading to reduced focus, motivation, and lower work engagement. Technology self-efficacy functions as a critical personal resource, buffering the impact of these demands. Employees who possess confidence in their technological skills are less prone to feeling overwhelmed and tend to perceive workplace challenges as manageable (4). According to the JDR model, personal resources like self-efficacy can assist in balancing or offsetting the load from job demands. This not only helps reduce exhaustion but also supports higher levels of engagement (61, 62). Consistent with that, the Conservation of Resource (COR) theory, Hobfoll (52), indicates that high personal resources, such as self-efficacy, help prevent the loss of psychological and cognitive resources, protecting employees from the downward spiral of exhaustion. Personal resources can help employees to buffer the effects of high job demands and maintain higher levels of engagement despite uncertainty and pressure imposed by technology adoption.

Although previous studies have consistently demonstrated that technostress negatively influences employee wellbeing and work engagement [(101), 60 and 26], less attention has been given to understanding the psychological processes through which these effects occur and the personal resources that may protect employees against technology-induced strain. Existing studies have largely examined technostress as a direct antecedent of adverse work outcomes, while overlooking the simultaneous roles of exhaustion as an explanatory mechanism and technology self-efficacy as a protective personal resource. Integrating the Job Demands Resource Model and the Conservation of Resource theory in the study helps to develop and test a model in which technology self-efficacy functions as a strategic personal resource that attenuates the depletion process initiated by technostress. In doing so, the study extends the JD-*R* model and the COR theory to the context of digital work by indicating how personal technological resources shape employees' responses to technology related job demands. This contributes to the technostress literature in three ways; firstly, it advances JD-*R* theory by demonstrating that technology self-efficacy should be conceptualized as a domain specific personal resource rather than merely a technological competence. Second, the study integrates the JD-*R* model and the COR theory to explain both the depletion process (technostress to exhaustion) and the protection process (technology self-efficacy to engagement). Finally, the findings provide evidence from a developing economy where rapid digital transformation has increased technological demands and this extends the generalizability of existing technostress theory beyond the Western contexts.

## Methodology

This study follows a positivist paradigm, which considers reality as objective, measurable, and accessible through reason and observation (63). This approach focuses on empirical data to test hypotheses and uncover relationships between variables (64). This study employed a quantitative research approach, which relies on empirical, numerical data to investigate relationships between variables (65). Quantitative methods were selected due to their structured approach, testability, and ability to facilitate analysis of statistical relationships using structural equation modeling as well as descriptive and inferential statistics (66). Specifically, the method was suited to test whether technology self-efficacy acts as a buffer against technostress and whether it helps sustain work engagement. The study followed a cross-sectional survey design to explore the relationships between technology self-efficacy, technostress, exhaustion, and work engagement. Given the quantitative nature, a descriptive approach was applied to observe and portray these variables as they occur in real-world settings (64, 67). Data was collected through a structured Qualtrics survey distributed by links and QR codes.

### Sample

This study used non-probability convenience sampling to collect primary data from employees in South African banks. Due to accessibility issues and limited time, this method efficiently collected data from available and willing participants (68, 69). An online questionnaire received 223 initial responses. After removing responses with under 90% completion, the final sample size was 202. This sample was considered sufficient for the statistical analysis. Although convenience sampling may introduce certain biases, standard procedures were implemented to improve data reliability and integrity (70). In terms of gender, female participants, accounted for 54%, while 44% were males. Regarding age, those between the ages of 31–40 were in the majority, accounting for 34%, followed by those between the ages of 41–50, making up 26% of the sample. Additionally, in terms of education, a large number of the participants held a bachelor's degree, making up 39% while those with PhDs contributed to only 1%. The sample is diverse in terms of the job positions of participants, with those in mid-level positions making up 55%, followed by those in entry-level positions accounting for 28%. Furthermore, the most common average hours spent using technology at work were those between 6 and 8 h (38%), followed by 4 and 6 h (37%), and above 8 h made 21% whilst the least amount accounted for 1–2 h (4%).

### Measures

The questionnaire included four sections: section A collected demographic data, and sections B to D contained validated scales measuring technostress, technology self-efficacy, work engagement and exhaustion. An explanation of the scales is provided below.

### Work engagement scale

Work engagement was assessed with the Utrecht Work Engagement Scale (UWES) by Schaufeli et al. (31). The scale consists of 17 items covering three dimensions: vigor (six items), dedication (five items), and absorption (six items). A 7-point Likert scale (0 = never, 6 = always) was used to evaluate responses. The UWES is a reliable measure of work engagement used globally (71). It demonstrates strong internal consistency, with reliability coefficients between 0.80 and 0.90, and has shown both concurrent and convergent validity in previous studies (72).

### Exhaustion scale

Exhaustion was measured with the Work Exhaustion Scale and Job Burnout Scale. Work exhaustion was assessed using five items from Moore (73), previously validated by Shih et al. (74), rated on a 7-point Likert scale. The scale showed solid reliability through both Cronbach's alpha and composite reliability. Job burnout was measured using 14 items from the Shirom-Milamed Burnout Measure, covering; Physical fatigue (six items; e.g., “I feel physically drained”); Emotional exhaustion (three items; e.g., “I feel I am unable to be sensitive”); Cognitive weariness (five items; e.g., “I feel I'*m* not thinking clearly”) (75). The overall scale demonstrated strong internal consistency (Cronbach's α = 0.96).

### Technostress scale

Technostress was assessed with Tarafdar Technostress Questionnaire Tarafdar et al. (1). The scale includes 21 items spanning five dimensions. Participants responded on a 7-point Likert scale, with higher scores indicating more intense technostress. The scale has shown strong internal consistency in previous studies, with Cronbach's alpha values above 0.80 for all subscales (26, 76), along with confirmed convergent and discriminant validity.

### Technology self-efficacy scale

Technology self-efficacy was assessed utilizing the modified Computer Self-Efficacy Scale (mCSES), originally developed by Compeau and Higgins (6) and subsequently adapted by Laver et al. (77). The scale includes 10 items, where participants rated their confidence in using technology on a 10-point Likert scale, ranging from 1 (“Not at all confident”) to 10 (“Completely confident”). This scale has proven to be reliable and widely applicable across various contexts, with a Cronbach's alpha of 0.94, indicating excellent internal consistency (77).

### Research procedure and ethical considerations

The ethical clearance and permission for the research were obtained from the University of Pretoria in the Economic and Management Sciences Faculty, reference- EMS221/23. The data collection process commenced only after receiving ethical clearance from the Ethics Committee in 2023. Participants signed a written informed consent and were fully informed of their rights, the voluntary nature of the study, and their ability to withdraw at any time without negative consequences through a detailed consent form. Data collection complied with the POPI Act (2013/2021), with no identifiable personal information gathered, and responses were securely submitted and encrypted. The research was conducted with integrity, objectivity, and strict adherence to ethical guidelines. Questionnaires were distributed via a Qualtrics link shared through the emails of consenting employees; follow-ups were done by the researcher to remind them to complete the questionnaire.

### Common method bias

To assess the potential influence of common method bias (CMB), a full collinearity assessment was conducted using the variance inflation factor (VIF) approach in SmartPLS, as recommended by Kock (78). According to this approach, VIF values below 3.3 indicate that common method bias is unlikely to pose a threat to the validity of the findings. The results showed that all inner model VIF values ranged from 1.000 to 2.395, which are in line with the recommended threshold of 3.3. These findings suggest that common method bias was not a significant concern in the study.

### Results

The data collected via Qualtrics was exported into Excel and SPSS in.sav formats. Following standard data cleaning procedures in SPSS version 29, incomplete responses, specifically participants who answered less than 90% of the survey, were removed, reducing the sample from 223 to 202 valid cases. To test the hypothesized relationships, Structural Equation Modeling (SEM) was conducted using SmartPLS (v4.1.0.8). This variance-based method was chosen for its suitability with small-to-moderate sample sizes and complex models (79). The SEM analysis followed a two-stage process. The outer mode, assessed the reliability and validity of constructs using composite reliability, average variance extracted (AVE), and indicator loadings. Acceptable thresholds included AVE ≥ 0.5 and loadings ≥ 0.7 (80). Structural model, the inner model, evaluated path coefficients (β), *p*-values, and *R*^2^ values to test the strength and significance of relationships between constructs (5).

In addition to the main model, a mediation analysis was performed to examine whether technology self-efficacy mediates the relationship between technostress and work engagement. Significance of indirect effects was tested within the inner model using bootstrapping (81). A moderation analysis tested whether technology self-efficacy buffers the effects of both technostress and exhaustion on work engagement. Interaction terms were evaluated in SmartPLS, and their significance was determined through path coefficients, *F*^2^ values, the effect size, and changes in *R*^2^. Together, these analytical procedures ensured a comprehensive understanding of the relationships between the study's core variables. The results for the outer model are presented below;

### Validity and reliability assessment

Confirmed from Table 1 below, all the study variables, along with their dimensions, met the internal consistency criteria, demonstrating excellent internal consistency reliability, meeting the quality criteria.

**Table 1:** Reliability Tests Results.

Variable	Number of items	Cronbach's alpha
Work engagement	17	0.980
Absorption	6	0.947
Vigor	6	0.942
Dedication	5	0.935
Technostress	21	0.972
Techno-complexity	4	0.903
Techno-overload	4	0.906
Techno-insecurity	5	0.905
Techno-uncertainty	4	0.936
Techno-invasion	4	0.921
Exhaustion	17	0.983
Technology self-efficacy	10	0.984

Table 1: reliability test results shows Cronbach's reliability scores for the measurement scales. First and foremost, the composite reliability estimates of all the scales were well above the recommended threshold of 0.7 (67, 82). The three dimensions of the work engagement scale showed reliability scores that are considered excellent (Table 1) with absorption (0.947), vigor (0.942), and dedication (0.935). Similarly, the internal consistency scores of the dimensions of technostress were estimated and presented: 0.903 (Techno-complexity), 0.906 (Techno-overload), 0.905 (Techno-insecurity), 0.936 (Techno-uncertainty), and 0.921 (Techno-invasion), all of which are regarded as excellent. The composite scale for exhaustion has an internal consistency score of 0.984, regarded as excellent. Moreover, the technology self-efficacy scale demonstrated 0.984 for its composite reliability score, considered excellent.

### Assessing the outer model: quality criteria

To further establish the internal consistency reliability of the outer model, the composite reliability scores were considered (Table 2). From Table 2 below, it can be confirmed that all the constructs of the study present satisfactory composite reliability, showing estimates above the standard of 0.6 score. Exhaustion showed (0.982), technostress (0.957), work engagement (0.986), and technology self-efficacy (1.00) (67). Additionally, assessments of the Average Variance extracted (AVE) and the Heterotrait-monotrait ratio (HTMT) scores confirmed the individual discriminant and convergent validity of technostress, technology self-efficacy, exhaustion, and work engagement. For validity, the AVE scores of all the constructs indicated values above the recommended 0.5 (67, 80) as per (Table 2), which all confirmed an acceptable convergent validity. Additionally, convergent validity was assessed by examining AVE scores, as presented in Table 2. The reported AVE scores indicate scores well beyond the cut-off score of 0.5, exhaustion (0.933), technostress (0.819), and work engagement (0.959), implying that all the scales demonstrate an acceptable convergent validity.

**Table 2:** Quality criteria, table reliability, and validity.

Variable	Cronbach's alpha	Composite reliability rho_c	Average variance Extracted (AVE)
Exhaustion	0.976	0.982	0.933
Technostress	0.945	0.957	0.819
Work engagement	0.979	0.986	0.959
Technology self-efficacy	1.00	1.00	1.00

Confirming the internal reliability and convergent validity of the constructs above, the discriminant validity of the constructs was to be confirmed. To do this, the HTMT scores were observed, which are presented in Table 3, showing the scores of the discriminant validity. According to Hair et al. (80) scores less than 0.90 indicate good discriminant validity, and within the current study, all the variables of the study confirm this requirement as follows: Technology self-efficacy and Exhaustion (0.573), Technostress and Exhaustion (0.662), technostress and technology self-efficacy (0.397), work engagement and exhaustion (0.701), work engagement and technology self-efficacy (0.726), and work engagement and technostress (0.604).

**Table 3:** Heterotrait-monotrait ratio (HTMT) list of discriminant validity.

Variable	Heterotrait-monotrait ratio (HTMT)
Tech_SE ↔ Exhaustion	0.573
Technostress ↔ Exhaustion	0.662
Technostress ↔ Tech_SE	0.397
Work Engagement ↔ Exhaustion	0.701
Work Engagement ↔ Tech_SE	0.726
Work Engagement ↔ Technostress	0.604

Having confirmed above the internal reliability, convergent and discriminant validity of the constructs of the study, the next step was to determine if the indicators/dimensions of each construct demonstrate significant loadings within their corresponding construct. Table 4 below presents the results of each of the loadings of the indicators of their constructs. It can be confirmed from Table 4 that all the dimensions indeed demonstrate statistically significant loadings with their constructs at *p* = *0.000*. The loadings of the work engagement construct ranged from 0.973 to 0.985, technostress (0.832 to 0.945) and exhaustion (0.954 to 0.972). It is worth noting that all the loadings show values above the recommended cut-off of 0.7 (5), confirming the quality criteria relating to the outer model, showing that the constructs of the study in the theoretical model are indeed valid and reliable.

**Table 4:** Outer loadings of constructs.

Variables	Original sample (O)	Sample mean (*M*)	Standard deviation (STDEV)	*T* statistics (|O/STDEV|)	*P* values
Absorption ← Work engagement	0.979	0.979	0.004	233.017	0.000
Cognitive_weariness ← Exhaustion	0.954	0.953	0.008	118.842	0.000
Dedication ← Work engagement	0.973	0.973	0.009	113.830	0.000
Emotional exhaustion ← Exhaustion	0.970	0.970	0.005	191.162	0.000
Physical fatigue ← Exhaustion	0.966	0.966	0.005	204.192	0.000
Tech SE ← Tech_SE	1.000	1.000	0.000	*n*/a	*n*/a
Techno_overload ← Technostress	0.945	0.945	0.010	98.414	0.000
Techno_complexity ← Technostress	0.937	0.937	0.008	120.084	0.000
Techno_insecurity ← Technostress	0.923	0.923	0.012	80.253	0.000
Techno_invasion ← Technostress	0.881	0.880	0.021	42.393	0.000
Techno_uncertainty ← Technostress	0.832	0.831	0.033	25.426	0.000
Vigor ← Work engagement	0.985	0.985	0.003	337.090	0.000
Work_Ex ← Exhaustion	0.972	0.972	0.004	249.999	0.000

In summing assessments of the outer model, one can confirm the reliability and validity of the constructs in the current model of the study. Hence, in the next phase of the study, the structural model /inner model of the study was evaluated by focusing on the conceptual model relating to the anticipated paths.

### Evaluation of the inner model

Prior to evaluating the structural relationships, collinearity among the predictor constructs was assessed using the variance inflation factor (VIF), this is important since high levels of multicollinearity can inflate standard errors, destabilize path coefficient estimates and complicate the interpretation of the structural relationships (79). In addition, following Kock (78), the full collinearity VIF approach was used to assess the potential influence of common method bias. As shown on Table 5, all VIF values ranged from 1.000 to 2.395, which are below the recommended threshold of 3.3. This indicates that multicollinearity was not a concern in the structural model and provide evidence that common method bias is unlikely to have materially influenced the study findings. The absence of problematic collinearity supports the stability and interpretability of the estimated structural relationships, thereby increasing confidence in the subsequent hypothesis testing.

**Table 5:** Collinearity statistics (VIF)-inner model.

Variables	VIF	Interpretation
Exhaustion → Work engagement	2.246	Acceptable
Technology Self-efficacy → Work engagement	1.907	Acceptable
Technology Self-efficacy x Exhaustion → Work engagement	2.322 →	Acceptable
Technology Self-efficacy x Technostress → Work engagement	2.395 →	Acceptable
Technostress → Exhaustion	1.000	Acceptable
Technostress → Technology Self-efficacy	1.000	Acceptable
Technostress → Work engagement	2.039	Acceptable

The path coefficients were examined to report the strength and direction of the proposed theoretical model. The study model has five direct paths. Table 6 presents path coefficients of the inner model, which are identified in the table by *p*- and *t*-values. Of the proposed direct paths to the dependent variable, all the direct paths are statistically significant (*p* < 0.05). The path from technostress to work engagement (β = −0.320:*t* = 4.037:*M* = −0.318: *p* = 0.000) showed a significant negative relationship, meaning technostress exhibits a strong negative influence on work engagement, providing support for Hypothesis 1: technostress negatively influences work engagement. Furthermore, the pathway from technostress to work exhaustion showed the strongest positive significant relationship (β = 0.645:*t* = 0.044, *M* = 0.646: *p* = 0.000), backing Hypothesis 2: Technostress positively influences exhaustion. The least significant relationship was observed between exhaustion and work engagement (β = −0.247 −0.247: *M* = 2.856: *p* = 0.003), indicating a significant negative relationship between exhaustion and work engagement, thus exhaustion negatively influences work engagement. Additionally, a positive statistically significant relationship was observed on the pathway from the exogenous variable, technology self-efficacy, to the endogenous variable, work engagement (β = 0.355:*M* = 0.357:*t* = 5.467: *p* = 0.000). Also, technostress and technology self-efficacy exhibited a significant negative relationship (β = −0.407:*M* = −0.407:*t* = 7.194: *p* = 0.000).

**Table 6:** Path coefficients of the inner model.

Path	Original sample (O)	Sample mean (*M*)	Standard deviation (STDEV)	*T* statistics (|O/STDEV|)	*P* values
Exhaustion → Work engagement	−0.247	−0.247	0.086	2.856	0.004
Tech_SE → Work engagement	0.355	0.357	0.065	5.467	0.000
Technostress → Exhaustion	0.645	0.646	0.044	14.717	0.000
Technostress → Tech_SE	−0.407	−0.407	0.057	7.194	0.000
Technostress → Work engagement	−0.320	−0.318	0.079	4.037	0.000
Tech_SE x Exhaustion → Work engagement	−0.055	−0.058	0.086	0.639	0.523
Tech_SE x Technostress → Work engagement	0.294	0.293	0.080	3.680	0.000

Table 7 shows the *R*-squared results of the variance of the dependent variables, indicating that the total of the independent variables explains 70.6% of the variance in work engagement and 41.6% in exhaustion.

**Table 7:** *R*-squared assessment for the inner model.

Variables	*R*-square	*R*-square adjusted
Exhaustion	0.416	0.414
Tech_SE	0.166	0.162
Work engagement	0.706	0.699

The structural model was evaluated in terms of collinearity, model fit, explanatory power and predictive relevance. As reported in Table 7 above, the model explained 41.6% of the variance in Exhaustion, 16.6% of the variance in Technology Self-Efficacy, and 70.6% of the variance in Work Engagement. Table 8 below shows that the Standardized Root Mean Square Residual (SRMR) was 0.053, indicating good model fit. Furthermore, all endogenous constructs yielded positive Q^2^ values, demonstrating adequate predictive relevance as shown on Table 8.

**Table 8:** Structural model assessment (SRMR, Q^2^, VIF/CMB).

Criterion	Value	Threshold	Good model fit
SRMR	0.053	< 0.08	Predictive relevance established
Q^2^-Exhaustion	0.410	>0	Predictive relevance established
Q^2^-Technology self-efficacy	0.158	>0	Predictive relevance established
Q^2^-Work engagement	0.323	>0	Predictive relevance established
Highest inner VIF	2.395	< 3.3	No multicollinearity; CMB unlikely

The effect sizes (*f* ^2^) were examined to determine the relative contribution of each predictor construct to the endogenous variables and the results are presented on Table 9. Guided by Cohen's (83) guidelines Technostress exerted a large effect on Exhaustion (*f* ^2^ = 0.714). Technology self-efficacy had a medium effect on work engagement (*f* ^2^ = 0.225), while Technostress observed medium effects on both Technology self-efficacy (*f* ^2^ = 0.199) and work engagement (*f* ^2^ = 0.171). Exhaustion had a small effect on work engagement (*f* ^2^ = 0.092), and the interaction between technology self-efficacy and technostress showed a small effect (*f* ^2^ = 0.116). In contrast, the interaction between technology self-efficacy and exhaustion produced a negligible effect (*f* ^2^ = 0.004).

**Table 9:** Effect sizes (*f* ^2^).

Structural relationship	*f* ^2^	Interpretation
Technostress → Exhaustion	0.714	Large
Technology Self-efficacy → Work engagement	0.225	Medium
Technostress → Technology self-efficacy	0.199	Medium
Technostress → Work engagement	0.171	Medium
Exhaustion → Work engagement	0.092	Small
Technology self-efficacy × Technostress → Work engagement	0.116	Small
Technology self-efficacy × Exhaustion → Work engagement	0.004	Negligible

To evaluate the hypothesis relating to mediation, the specific indirect effects were considered. As such, Table 10 presents the degree to which technology self-efficacy mediates the relationship between technostress and work engagement. From the statistical evidence, one can confirm that technology self-efficacy does indeed have a significant mediating effect on the relationship between technostress, and work engagement (β = −0.144:*M* = −0.145:*t* = 4.273, *p* = 0.000). Hence, the mediation results support Hypothesis 3. However, it is worth noting that this is a partial mediation since the path coefficient between technostress and work engagement is significant too, as can be confirmed from Table 6. Additionally, it is evident in Table 10 that exhaustion also partially mediates the relationship between technostress and work engagement (β = −0.159:*M* = −0.159:*t* = 2.810, *p* = 0.005).

**Table 10:** Specific indirect effect for evaluation of the mediation effect.

Indirect paths	Original sample (O)	Sample mean (*M*)	Standard deviation (STDEV)	*T* statistics (|O/STDEV|)	*P*-values
Technostress → Tech_SE → Work engagement	−0.144	−0.145	0.034	4.273	0.000
Technostress → Exhaustion → Work engagement	−0.159	−0.159	0.057	2.810	0.005

**Figure 2:**
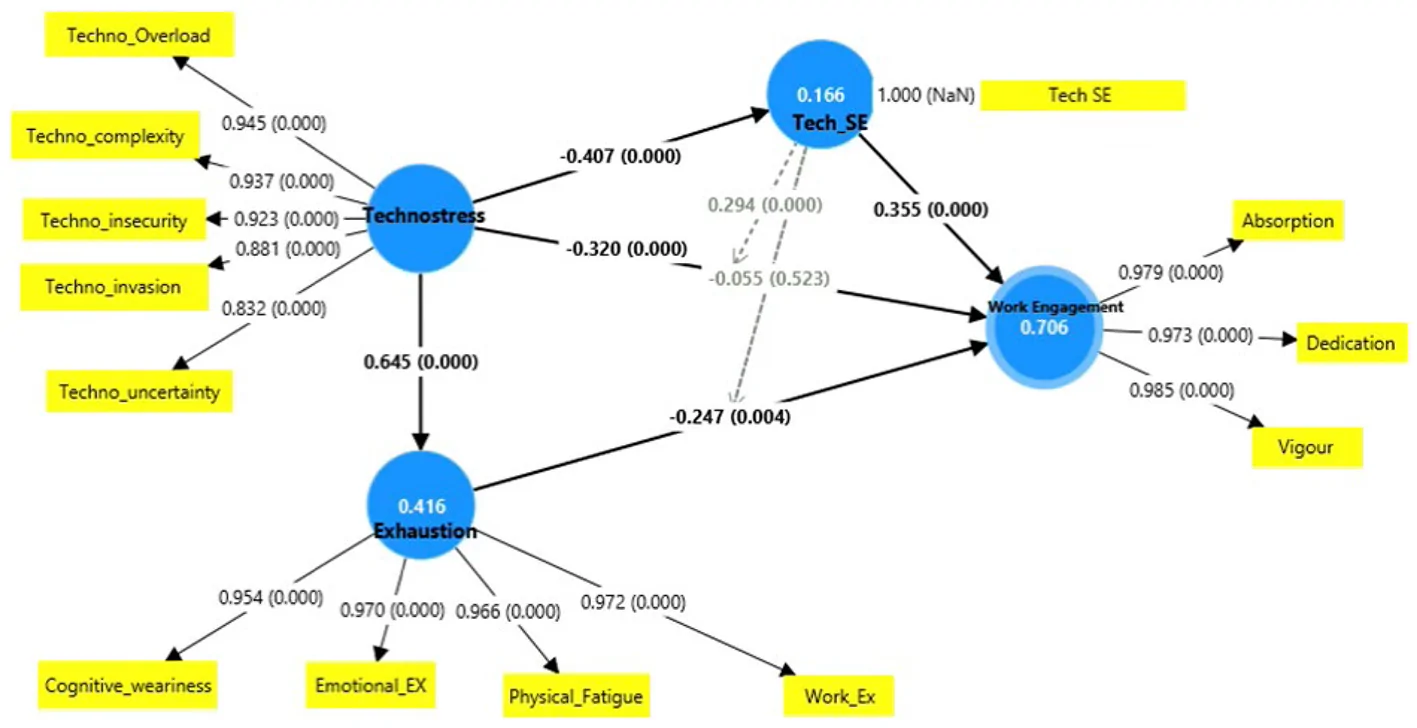
Mediation model. A mediation model.

Figure 2 below shows the mediation model of the study.

To evaluate the hypothesis regarding the moderation effect of technology self-efficacy, Table 11 presents path coefficients of the inner model, calculated on bootstrapping involving complete slower, one-tailed testing with a 0.05 level of significance, and bias-corrected and accelerated BCa bootstrapping.

**Table 11:** Path Coefficients for the moderation analysis.

Moderation path	Original sample (O)	Sample mean (*M*)	Standard deviation (STDEV)	*T* statistics (|O/STDEV|)	*P*-values
Exhaustion → Work engagement	−0.247	−0.247	0.086	2.856	0.002
Tech_SE → Work engagement	0.355	0.357	0.065	5.467	0.000
Technostress → Exhaustion	0.645	0.646	0.044	14.717	0.000
Technostress → Tech_SE	−0.407	−0.407	0.057	7.194	0.000
Technostress → Work engagement	−0.320	−0.318	0.079	4.037	0.000
Tech_SE x Exhaustion → Work engagement	−0.055	−0.058	0.086	0.639	0.261
Tech_SE x Technostress → Work engagement	0.294	0.293	0.080	3.680	0.000

The moderation effect can be identified by interaction terms (Tech_SE x technostress) or (Technostress x exhaustion) that have a significant impact on the endogenous variable (work engagement). Accordingly, as shown on Table 11 the interaction term Tech_SE x technostress → work engagement showed that technology self-efficacy moderates the relationship between technostress and work engagement (β = 0.294:*M* = 0.293:*t* = 3.680: *p* = 0.000). In addition, since technology self-efficacy has a direct, positive, and significant effect on work engagement (β = 0.355; *p* = 0.000), the moderation is an amplifying one. Hence, Hypothesis 5b is not rejected: technology self-efficacy moderates the relationship between technostress and work engagement.

**Figure 3:**
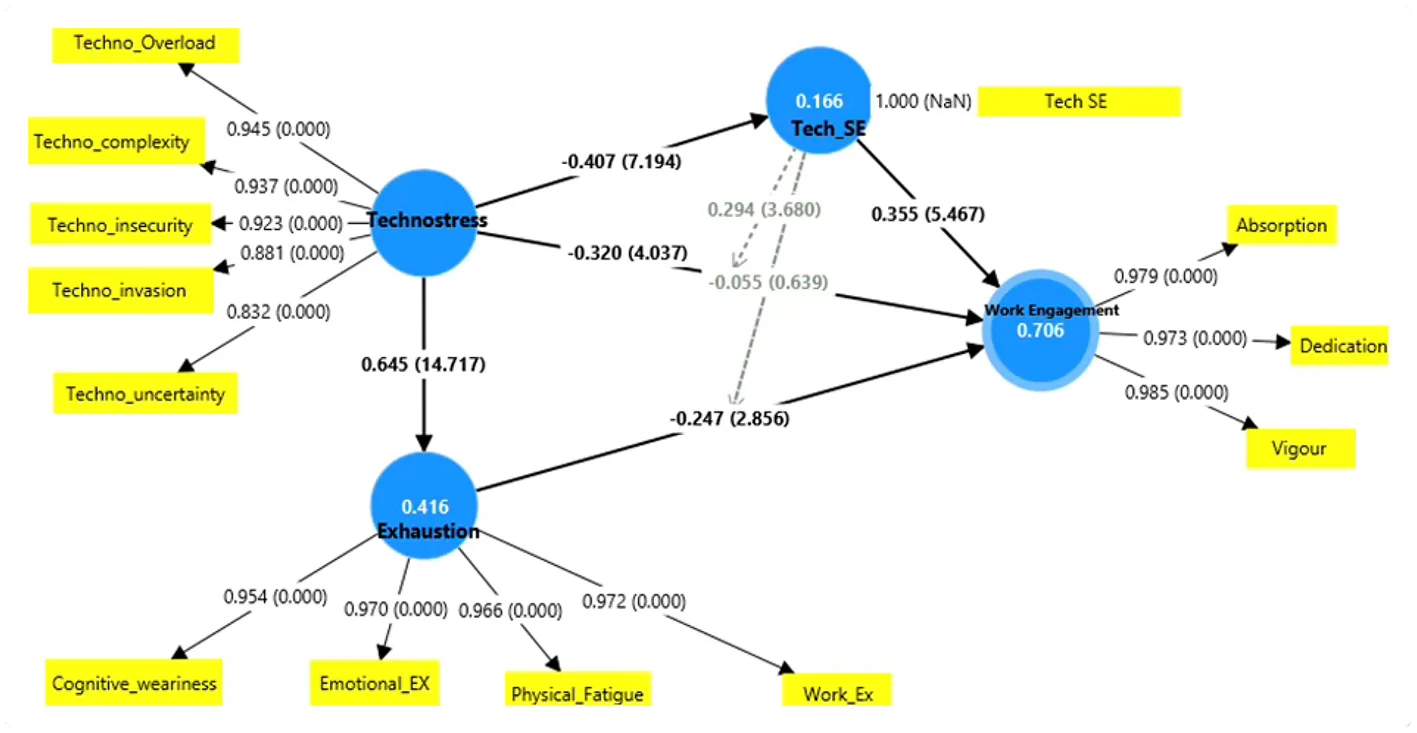
Moderation model. In this figure, path coefficients and *t*-values are presented, and the constructs show *R*-square values.

However, evaluating the moderation effect of technology self-efficacy in the relationship between technostress and exhaustion did not yield statistically significant results (β = −0.055:*M* = −0.058, *t* = 0.639, *p* = 0.261). Therefore, hypothesis 5a is rejected. Figure 3 presents the moderation model of the study.

Having established the moderating effect of technology self-efficacy, to understand the extent of the moderating effect, the calculation of the moderating effect size (*F*-squared effect size) was deemed important (79). Interpretations: as per (84), guidelines for interpreting f^2^-effects, showing small (0.005), medium (0.01), and large effects (0.025).

Table 12 presents the effect size of the moderation effect of technology self-efficacy in the relationship between technostress and work engagement. As evidenced on Table 12 below, the moderation analysis revealed that the interaction between technology self-efficacy and exhaustion produced a negligible effect size (*f* ^2^ = 0.004), which was not statistically significant (*t* = 0.212, *p* = 0.416). This finding indicates that technology self-efficacy did not meaningfully influence the relationship between exhaustion and work engagement. In contrast, the interaction between technology self-efficacy and technostress yielded an effect size of (*f* ^2^ = 0.115), indicating a modest practical contribution to explaining work engagement. However, the bootstrapped significance test for this effect narrowly missed the conventional 5% significance level (*t* = 1.639, *p* = 0.051). Although this result suggests that technology self-efficacy may play a buffering role in the relationship between technostress and work engagement, the practical contribution of the moderation effect should be interpreted with caution. Findings provide some evidence that technology self-efficacy could slightly play a protective role in maintaining engagement under conditions of technostress.

**Table 12:** Effect size of the moderation effect.

Variables	Original sample (O)	Sample mean (*M*)	Standard deviation (STDEV)	*T* statistics (|O/STDEV|)	*P-*values
Tech_SE x Exhaustion → Work engagement	0.004	0.014	0.018	0.212	0.416
Tech_SE x Technostress → Work engagement	0.115	0.126	0.070	1.639	0.051

## Discussion

The focus was on five hypotheses: the direct influence of technostress on work engagement, the direct influence of technostress on exhaustion, the mediating role of technology self-efficacy (TSE) in the relationship between technostress and work engagement and the mediating role of technology self-efficacy in the relationship between technostress and exhaustion. The study also focused on the moderating role of TSE in the relationship between exhaustion and work engagement, and the moderating role of TSE between technostress and work engagement.

The results indicated that technostress negatively influences work engagement (β = −0.320:*t* = 4.037:*M* = −0.318, *p* = 0.000), suggesting that increasing technology related demands deplete employees' psychological resources and reduce their motivation to remain engaged. The observed finding supports the JD-*R* model which proposes that excessive job demands trigger a health impairment process that undermines work engagement (55). As employees face high technology-related job demands, their energy and motivation decline, leading to lower enthusiasm, lower dedication, and lower absorption in work tasks. Technostress acts as a drain on resources, reducing employees' capacity to stay engaged. The findings confirm prior studies, such as Di Dalmazi et al. (85), which show that strains of technostress reduce employees' performance and diminish their work engagement, also technostress diminishes employee wellbeing and engagement in digitally intensive work environments (3, 5, 26). In line with the JD-*R* framework, technostress as a job demand, through its five technostress creators negatively impacts work engagement. Similarly, Salo et al. (86) found that techno-uncertainty has an adverse impact on work engagement, particularly in contexts like banking, with constant ICT upgrades creating pressure and skill mismatches (17). Moreover, studies such as Marchiori et al. (87) show the inevitable negative effect of technostress (i.e., techno-invasion) arising from hybrid, remote work, causing misunderstanding at work, blurring boundaries, affecting the quality of life, productivity, wellbeing, and work engagement (88).

The results also confirm hypothesis 2, which proposes that technostress is positively related to work exhaustion (β = 0.645, *p* < 0.001). This suggests that prolonged exposure to technology related demands progressively depletes employees' cognitive and emotional resources, increasing the likelihood of exhaustion. As technostress increases, employees are more likely to feel drained, fatigued and emotionally exhausted. Consistent with these findings, Shen and Kuang (49) highlighted that prolonged exposure to workplace ICT changes and new systems depletes energy and psychological resources, leading to exhaustion. Persistent technological demands contribute to fatigue, burnout and reduced wellbeing (4, 26). Similarly, Shen and Kuang (49) noted that sustained exposure to new technology led to constant uncertainty and complexity, eventually causing depersonalization, whereby employees develop negative attitudes toward their jobs, potentially heightened by the constant need to adapt to new technologies. Shen and Kuang (49) also identified burnout, especially a diminished sense of personal accomplishment, as a significant outcome of technostress. Overall, the findings of the study are in agreement with existing literature, which demonstrates that technostress significantly contributes to work exhaustion, as employees under such stress often experience heightened emotional, cognitive, and physical fatigue, consistent with similar findings from Gemmano et al. (50) and Buenadicha-Mateos et al. (89).

The results also indicated that technology self-efficacy does indeed have a significant mediating effect on the relationship between technostress and work engagement (β = −0.144:*M* = −0.145:*t* = 4.273, *p* = 0.000). This suggests that when employees experience high levels of technostress, their confidence in using technology decreases, which in turn lowers their engagement, thus technostress reduces engagement partly by undermining employees' confidence in managing technological demands. This finding supports Social Cognitive Theory (102) and is consistent with evidence that technology self-efficacy facilitates adaptation to digital work environments (4, 7). However, the findings should be interpreted alongside studies that have emphasized the importance of organizational context. For example, Harunavamwe and Kanengoni (5) found that perceived organizational support played a critical role in buffering the effects of technostress, while Molino et al. (90) demonstrated that job resources, such as training and supervisory support, significantly reduce technostress. Similarly, Tarafdar et al. (26) argued that technological demands may be experienced as opportunities for learning rather than sources of strain when employees receive adequate organizational support. These differences suggest that the influence of technology self-efficacy may depend on the availability of complementary organizational resources, highlighting the importance of combining individual capability development with supportive workplace practices. This supports the notion that employees with stronger technology self-efficacy are better able to cope with technostress, thereby sustaining engagement (26, 38). However, because the direct path between technostress and work engagement remains significant, this mediation is only partial, meaning technostress continues to directly undermine engagement even after accounting for self-efficacy. The findings are consistent with previous studies like Llorens et al. (91), who confirmed the mediating role of technology self-efficacy in the relationship between technostress and burnout, which is denoted as the inverse of work engagement.

Surprisingly, the findings indicated that technology self-efficacy does not have a significant mediating effect on the relationship between technostress and exhaustion. Self-efficacy alone may not be sufficient to offset the resource-draining effects of technostress on employees' energy levels. Even though technology self-efficacy can help employees feel more capable of handling technological demands, it does not necessarily prevent the cognitive and emotional strain that leads to exhaustion (92). Employees with high self-efficacy might even take on more technological tasks, which could sustain or worsen fatigue rather than buffer it (58).

Interestingly, results indicated that exhaustion partially mediates the relationship (β = −0.159, *p* = 0.005), indicating that technostress contributes to feelings of fatigue and depletion, which subsequently reduce work engagement. This aligns with the Conservation of Resources (COR) theory, which posits that resource loss (in this case, energy and coping ability) is a major driver of stress outcomes (93). These findings suggest that while enhancing employees' technology self-efficacy can buffer some of the negative impact of technostress, addressing exhaustion is equally critical to maintaining engagement.

The results did not support (Hypothesis 5a) that technology self-efficacy moderates the relationship between technostress and exhaustion (β = −0.055:*M* = −0.058, *t* = 0.639, *p* = 0.261). This suggests that confidence in using technology does not necessarily shield employees from the energy-draining effects of technostress. This maybe because technology self-efficacy enhances employees' confidence in using technology but may not offset chronic job demands, excessive workloads or inadequate organizational support. Contrary to the current findings, previous studies (27) indicated that technology self-efficacy buffers the effects of technostress on burnout and emotional exhaustion. Though self-efficacy can enhance task performance and reduce feelings of inefficacy, it may not prevent the emotional and cognitive fatigue associated with prolonged technology demands (58). Even though technology self-efficacy as a personal resource may aid employees to feel confident in their capacity to use technology (94, 95), it may not be robust enough to mitigate the adverse effects of exhaustion. Exhaustion, as a negative work outcome in the JD-*R*, represents a state of extreme emotional and physical fatigue and cognitive depletion (89). From the COR theory, exhaustion occurs when resource loss outweighs resource gain. As argued by Tarafdar et al. (26) and Pansini et al. (7), the effects of technostress are shaped by the interaction between personal and organizational resources and reducing exhaustion requires not only improving employees' technological confidence but also addressing workplace factors such as workload, digital support, and job design. Though technology self-efficacy may help employees cope with technical challenges, it does not replenish the psychological and physical resources depleted by persistent technostress.

Furthermore, previous studies show that the buffering role of technology self-efficacy might be more pertinent in averting the onset of technostress and aiding employees in coping with technical challenges (54) rather than alleviating exhaustion once it has set in. This is because once employees are emotionally and physically depleted, personal resources (technology self-efficacy) may not be sufficient ground to foster work engagement, which is manifested in vigor, dedication, and absorption at work (48). This indicates that factors such as recovery opportunities, organizational support, and workload management may play a more critical role in buffering exhaustion than self-efficacy alone (96).

The results confirmed that technology self-efficacy moderates the relationship between technostress and work engagement (β = 0.294:*M* = 0.293:*t* = 3.680: *p* = 0.000), suggesting that employees with higher levels of technology self-efficacy are better able to maintain or even enhance their work engagement despite experiencing technostress. This signifies that when individuals feel confident in their ability to use technology effectively, the negative impact of technostress on engagement is weakened. The findings align with the JD-*R* model (55), which posits that personal resources play a role in buffering the strain of high demands while sustaining motivation and engagement. Technology self-efficacy enhances adaptability and resilience in digital work environments, enabling employees to cope more effectively with overload, complexity, and uncertainty (26, 97).

Note is taken that the moderation effect size (f^2^ = 0.115: *p* = 0.051) is marginally non-significant, narrowly missing the conventional threshold (*p* < 0.05), suggesting a potential interaction that warrants further investigation. The findings suggest that technology self-efficacy is more relevant in mitigating the effects of technostress than exhaustion on work engagement. Although the moderation effect demonstrated a modest effect size (*f* ^2^ = 0.115), its bootstrapped significance was marginal (*p* = 0.051), indicating that the protective influence of technology self-efficacy should be interpreted with caution. Nevertheless, the findings are consistent with Social Cognitive Theory (102) and the Job Demands–Resources (JD-*R*) model (55), which posit that personal resources enable employees to cope more effectively with demanding work conditions. Similarly, Rohwer et al. (4) and Pansini et al. (7) found that technology self-efficacy and related personal resources can reduce the adverse effects of technostress, although their effectiveness is strengthened when supported by organizational resources. Recent evidence also indicates that organizational support plays a critical role in sustaining work engagement in technology-intensive work environments (5). This therefore imply that technology self-efficacy should be viewed as one component of a broader organizational strategy that includes technology training, leadership support, and manageable workloads. In contrast, the negligible and non-significant moderation effect for exhaustion (*f* ^2^ = 0.004, *p* = 0.416) indicates that technology self-efficacy does not buffer the relationship between exhaustion and work engagement. This finding is consistent with the Conservation of Resources (COR) theory, which suggests that once employees experience substantial resource depletion, personal resources alone may be insufficient to sustain engagement (103). Therefore, interventions aimed at reducing exhaustion should extend beyond enhancing technology self-efficacy and focus on reducing excessive job demands, promoting employee recovery, and strengthening organizational support (55). Additionally, both the mediation and moderation models yielded identical *R*^2^ values for Work Engagement. This implies that even though the models tested different structural pathways, the underlying predictors (technostress, technology self-efficacy, exhaustion, and their interaction) explain a similar proportion of variance in the outcome.

### Practical and theoretical implications

The results confirm that technostress not only diminishes work engagement but also heightens exhaustion, indicating the dual impact on both motivation and strain pathways. Given this, organizations need to adopt a two-pronged approach, first by reducing the sources of technostress through measures such as workload management, simplified IT systems and establishing digital boundaries. Secondly, organizations need to implement wellness and recovery initiatives such as rest breaks, resilience-building programs, and psychological support to help employees restore depleted resources and sustain their energy and engagement (26).

Both technology self-efficacy and exhaustion partially mediate the relationship between technostress and work engagement, suggesting that individual confidence with technology and their energy levels play a critical role in shaping how technostress translates into disengagement. This highlights the importance of training, mentoring and digital literacy programs within Human Resource development strategies. Exhaustion reflects the resource-depleting pathway through which technostress reduces engagement, emphasizing the need for proactive fatigue management through workload balancing, supportive leadership, and wellness initiatives. Bank managers should focus on building employees ‘technological confidence while simultaneously safeguarding their energy reserves, ensuring sustained engagement in technology-intensive workplaces.

Moderation results suggested that technology self-efficacy helps to sustain engagement but does not prevent exhaustion under technostress. Since the moderation results are consistent with theory and previous studies, where technology self-efficacy was proved to moderate the relationship between technostress creators and performance at work (62) and also moderate the relationship between technostress and burnout, which has been proven to be the antithesis of work engagement (27) organizations should therefore strengthen self-efficacy through digital training and mentoring to keep employees engaged while also reducing systematic stressors and supporting recovery to manage fatigue and prevent burnout. Organizations experiencing high technological changes should thus invest in continuous digital skills training to build employees ‘confidence in using technology effectively. Chang et al. (95) advised that consistent digital upskilling increases employees' intention to adopt new technologies at work.

In terms of theoretical implications, the results extend the JD-*R* model and align with the COR theory by demonstrating that technostress as a job demand reduces engagement through exhaustion, reflecting resource depletion, while technology self-efficacy, as a personal resource buffer, mitigates its negative effects on engagement but not on exhaustion (54). This highlights technostress as both a strain outcome (exhaustion) and a motivational inhibitor (reduced engagement). The results refine self-efficacy theory (98) by showing that technology self-efficacy protects engagement yet has domain-specific limitations in preventing resource loss. Though personal resources can help maintain engagement, they cannot fully compensate for the resource depletion caused by technostress.

### Limitations and future directions

The study examined employees in the banking industry; therefore, the findings from this study need to be tested in other contexts, considering that the experience of employees may differ depending on the type of organization as well as the nature of work. The study's cross-sectional design and reliance on self-reported data limit causal inferences and may introduce bias (99). Future research could adopt longitudinal or experimental designs, explore additional personal and organizational resources, and examine diverse work settings to better understand and mitigate technostress. Future studies may also focus on designing a follow-up survey to examine an overall perspective for assessing the effect of technostress, technology self-efficacy, and exhaustion on work engagement over time.

## Conclusion

The study demonstrates that technostress negatively influences work engagement and positively influences exhaustion. Technology self-efficacy mitigates the direct effects of technostress on engagement and partially breaks the stress-to-disengagement pathway. However, its buffering capacity weakens in the face of exhaustion, making a case for the need for organizational interventions that reduce technostress itself and not just build individual resilience. The findings of the study suggest that technology self-efficacy plays an important but nuanced role in sustaining engagement under technostress, emphasizing the need to consider both personal and contextual resources when addressing the impact of technostress in the workplace.

## Data Availability

The raw data supporting the conclusions of this article will be made available by the authors, without undue reservation.
